# Four converging measures of temporal discounting and their relationships with intelligence, executive functions, thinking dispositions, and behavioral outcomes

**DOI:** 10.3389/fpsyg.2015.00728

**Published:** 2015-06-05

**Authors:** Alexandra G. Basile, Maggie E. Toplak

**Affiliations:** Department of Psychology, LaMarsh Centre for Child and Youth Research, York UniversityToronto, ON, Canada

**Keywords:** temporal discounting, rational thinking, decision-making, intelligence, executive functions, thinking dispositions, behavioral outcomes

## Abstract

Temporal discounting is the tendency to devalue temporally distant rewards. Past studies have examined the *k*-value, the indifference point, and the area under the curve as dependent measures on this task. The current study included these three measures and a fourth measure, called the interest rate total score, which differentiated good from poor choices. The interest rate total score was based on scoring only those items in which the delayed choice should be preferred given the expected return based on simple interest rates. In addition, associations with several individual difference measures were examined including intelligence, executive functions (inhibition, working memory, and set-shifting), thinking dispositions [Need for Cognition and Consideration of Future Consequences (CFCs)] and engagement in substance use and gambling behavior. A staircase temporal discounting task was examined in a sample of 99 university students. Replicating previous studies, temporal discounting increased with longer delays to reward and decreased with higher reward magnitudes. A hyperbolic function accounted for more variance in temporal discounting than an exponential function. Reaction time at the indifference point was significantly longer than at the other choice points. The four dependent measures of temporal discounting were all significantly correlated and were also significantly associated with our individual difference measures. That is, the tendency to wait for a larger delayed reward on all of the temporal discounting measures was associated with higher intelligence, higher executive functions, and more CFCs. Associations between our measures of temporal discounting and outcomes related to substance use and gambling behavior were modest in our university sample.

## Introduction

Temporal discounting refers to the tendency to discount rewards that are temporally distant, where there is a weakening of the effects of value due to temporal delay (Critchfield and Kollins, 2001). Temporal discounting, also known as delay discounting, has become an index of self-control and willpower (Ainslie, 2001, 2003; Shamosh and Gray, 2008). It is a significant construct that has been conceptualized as prudently discounting the future in models of rational thinking and decision-making (Stanovich, 2009, 2011; Stanovich et al., 2011). There are several methodological considerations in the measurement of temporal discounting that spans across different literatures, including psychophysical methods (Mazur, 1987; Myerson et al., 2003) and adapted methods that have been used in the judgment and decision-making literature (Hardisty et al., 2013). The key dependent measures that have been examined in the temporal discounting literature are the *k*-value, area under the curve, and the indifference point. Given that temporal discounting has been conceptualized as a component of instrumental rationality (Stanovich, 2009, 2011), we included one additional indicator called the interest rate score, based on a scoring scheme that differentiated better from poorer choices. Our purpose was to examine whether these four temporal discounting measures would be significantly associated. In addition, we included individual difference measures of cognitive abilities (intelligence and executive functions) and thinking dispositions as further measures to examine convergence. Finally, we included a measure of behavioral outcomes to assess the association between temporal discounting and drug use and gambling behavior. Temporal discounting is an important construct that has been identified across several literatures, and this study contributes to our understanding of optimal ways to assess this construct with the use of a single indicator.

### Temporal Discounting

Temporal discounting tasks generally require participants to make choices between a small variable reward available immediately versus a larger constant reward available after a variable delay (Rachlin et al., 1991). These types of tasks have also been referred to as intertemporal choice and delay discounting, and sometimes even delay of gratification (Shamosh and Gray, 2008). However, some researchers in the judgment and decision-making field have made more nuanced distinctions between various considerations that underlie these choices, such as factors that diminish the expected utility of a future consequence (time discounting) and considerations that may lead to preference for immediate utility over delayed utility (time preference; Frederick et al., 2002). In this study, we focused on single indicators of performance on typical choice tasks, also called the “commitment-choice” procedure, which requires the individual to commit to an immediate or delayed reward on several different trials (Frederick et al., 2002; Reynolds and Schiffbauer, 2005).

**FIGURE 1:**
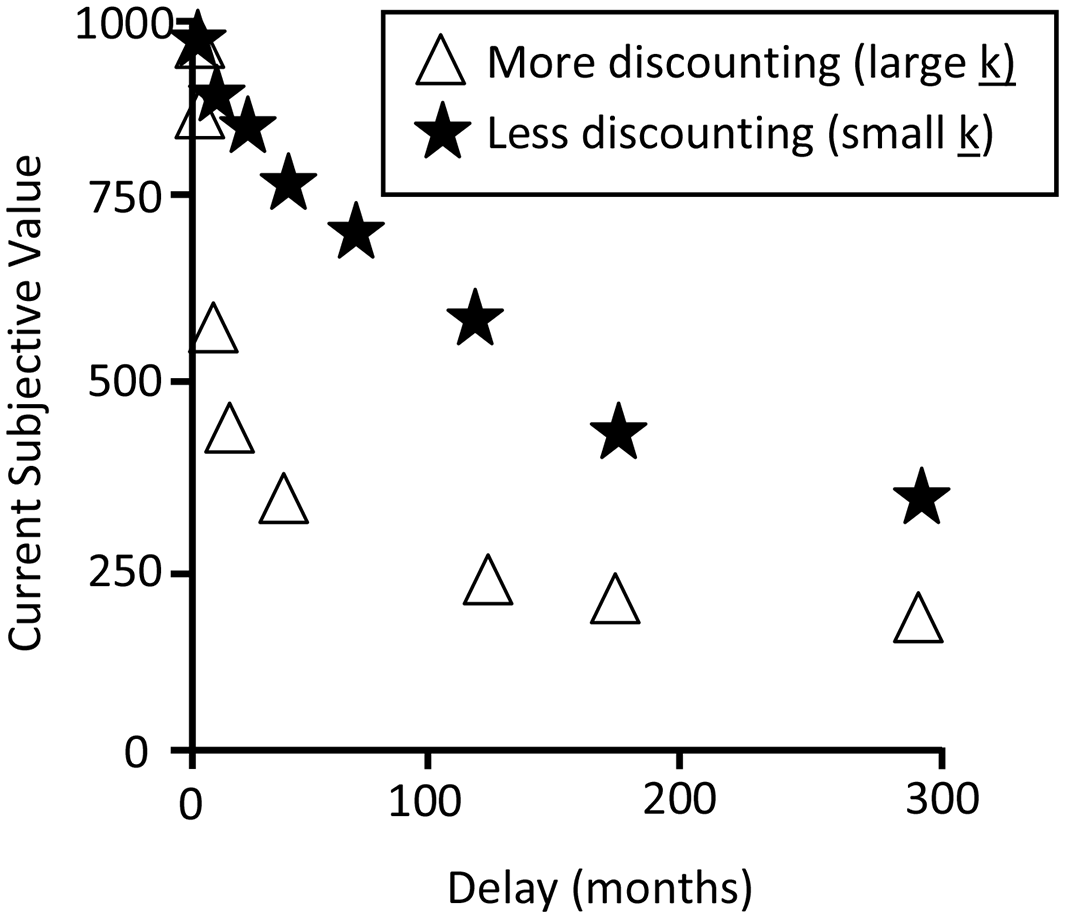
**Hypothetical temporal discounting data illustrating indifference point choices, based on Critchfield and Kollins (2001)**.

In temporal discounting tasks, there is an indifference point, where the participant will switch from preferring the immediate reward to the delayed reward. The indifference point represents the subjective value of the reward for the participant because it is the amount preferred (which will vary by participant) and is usually less than the face value of the larger delayed reward (Critchfield and Kollins, 2001). The data from this task can be represented graphically with the current subjective value of the reward on the *y*-axis and the delay on the *x*-axis. **Figure 1** contains hypothetical data from two participants to illustrate two different choice architectures.

The data in **Figure 1** can be represented by a hyperbolic or an exponential equation (Mazur, 1987):

$$\text{Hyperbolic}:V=\frac{A}{1+kD}$$

$$\text{Exponential}:V\;=\;Ae^{-kD}$$

*A*, *V*, and *D* represent the amount, subjective value, and delay respectively. Variables in the exponential equation represent the same values as those in the hyperbolic equation and *e* represents Euler’s number and is also at the base of the natural logarithm. A *k*-value can be derived, which represents an individual fitted parameter that can be thought of as sensitivity to delay. When the value of *k* is small, the individual is less sensitive to delay, and shows less discounting in response to it (a less steep curve, denoted by the stars in **Figure 1**). However, when the value of *k* is large, it means that the individual is very sensitive to the delay period, and this translates to higher rates of discounting in response to delay (a steep curve, denoted by the triangles in **Figure 1**). Indeed, much research involving temporal discounting data has found that the data is fit best with the hyperbolic equation, as opposed to the exponential equation (Rachlin et al., 1991; Green et al., 1997; Ainslie, 2001; Johnson and Bickel, 2002; Myerson et al., 2003; Robles and Vargas, 2007; Steinberg et al., 2009), meaning that individuals were discounting at a negatively accelerating rate. In order to measure people’s choice architecture in temporal discounting paradigms, the *k*-value is commonly used (Mazur, 1987).

The area under the curve is an additional dependent measure that is calculated by normalizing each delay and subjective value for each data point (Myerson et al., 2001). As noted by Myerson et al. (2001), since the area is calculated by the actual data points, there are no theoretical assumptions inherent in the area under the curve measure. The indifference point (*V*; Critchfield and Kollins, 2001) indicates when the participant switched from preferring the immediate reward to the delayed reward. For example, a participant may choose to wait 1 month for $100 over $50 now, but when given the choice between $60 now or $100 in 1 month, the participant may choose $60 now. This would indicate that $60 now is the indifference point or the subjective value of the delayed reward. All three of these commonly used measures to assess temporal discounting were examined in the present study. We tested whether a hyperbolic or exponential curve would provide a better fit of our data.

The hyperbolic function used to account for temporal discounting choices shows that discounting rates are not constant over time and appear to decline (Frederick et al., 2002). Indeed, length of delay has been identified as a critical variable. Green et al. (1997) found that the amount of the immediate reward that is subjectively equivalent to the later reward decreased with delay. Therefore, as delay increased, subjective value of a reward decreased. As time to reward increased, the reward itself is judged to be equivalent to smaller and smaller rewards. This finding was also supported by Steinberg et al. (2009) who demonstrated that the indifference point declines as the delay interval increases. The longer one has to wait for a reward, the less value that reward holds in comparison to the immediate reward being offered.

The rate at which the value of a delayed reward is discounted also depends on the magnitude or size of that reward. Green et al. (1997) reported that the delayed reward decreased in a negatively accelerating fashion as the amount of the reward increased from $100 to $25,000, but no differences in discounting were observed from $25,000 to $100,000. These findings provided evidence to suggest that the rate of discounting decreases with increases in the amount of delayed reward, up to a certain point (as measured by the *k* parameter). In other words, with increasing reward value, delay discounting tends to decrease. These results are consistent with Myerson et al. (2003) who found that smaller delayed amounts were discounted more steeply than larger delayed amounts. We included five periods of delay and two different reward magnitudes in the current study to test for the effects of period of delay and size of reward magnitude.

All of the measures of temporal discounting discussed thus far do not differentiate better from poorer choices in the calculation of the dependent variable. Temporal discounting can be conceptualized as a component of instrumental rationality, pertaining to an individual’s goal fulfillment (Stanovich, 2011). Temporal discounting, however, is a theoretically complex construct (Frederick, 2006), and some choices may be characterized as better decisions than others. While there are many contextual factors that can impact the immediate versus delayed choice, some of these choices are arguably better than others. In Aesop’s classic fable of the grasshopper and the ant, the ant maximized its individual goal fulfillment by toiling away to put food away for the winter, while the grasshopper wasted the opportunity to put away food and instead basked in the nice weather, only to find itself hungry once the winter season arrived (McClure et al., 2004). This fable has been used to characterize temporal discounting and it would be difficult to dispute that the ant made better judgments than the grasshopper.

In order to differentiate better from poorer choices, another way to assess temporal discounting is from the perspective of market interest rates (Senecal et al., 2012). For example, if offered $20 now versus $100 in 5 years, and $90 now and $100 in 5 years, the delayed choice in the former is a better option than the delayed choice in the latter. Indeed, the former provides an 80% annual rate of return based on a calculation of simple interest rates, and the latter provides a 2% rate of return. Another potential measure to index temporal discounting is to derive a score that only includes items in which the choice to wait for the larger delayed reward is arguably the best option. From the perspective of simple interest rates, the estimate of an annual interest rate could arguably be used to separate choices where one should wait relative to the remaining choices. For example, Frederick (2005) found that a five-item time preference test that credited participants with selecting the delayed later choice was significantly associated with performance on the cognitive reflection test and his finding also was replicated by Toplak et al. (2014). All of these five items on this time preference test would have translated into at least a 40% increase based on a simple interest rate basis. To this end, we scored only a subset of the temporal discounting items. In particular, we selected a 40% cut-off, a rate of return that would clearly reflect a poor judgment or decision to pass up. We credited participants if they made the delayed choice when a calculation of an annual interest rate was at or above a 40% rate of return. This was our fourth indicator of temporal discounting, which we called the interest rate total score. We characterized this measure as an indicator of decision-making given that we differentiate poor from better choices in the scoring of this task. Temporal discounting has been included in taxonomies of rational thinking and decision-making (Stanovich, 2009, 2011; Stanovich et al., 2011). We expected the findings from the interest rate total score to converge with the other indicators of temporal discounting.

If the shift from the immediate option to the larger delayed option involves an override of a default in favor of longer term optimization, then we may also expect differences in reaction time on the indifference point selection compared to other selections. Robles and Vargas (2007) reported significant peaks in reaction time at the first trial and at or about the indifference trial on a temporal discounting task that was administered using ascending and descending orders. We predicted that the reaction time would be highest at the indifference point relative to all of the other trials, given that this requires additional consideration in order to override the default choices on this task.

### Individual Differences and Temporal Discounting

Why are some people more willing to wait for large delayed rewards while others prefer smaller, more immediate, rewards? One common explanation has been cognitive abilities. Shamosh and Gray (2008) conducted a meta-analysis of the relationship between temporal discounting and intelligence. These authors found that people with higher intelligence displayed lower delay discounting or less sensitivity to delay periods. Steinberg et al. (2009) also found that indifference points were positively related to intelligence. Namely, participants with higher intelligence scores were more willing to wait for a larger delayed reward.

Executive functions are another domain of cognitive abilities that have been examined as a correlate of temporal discounting. Executive functions are typically assessed using measures of inhibition, working memory, and set-shifting (or mental flexibility). Inhibition refers to the ability to stop a prepotent response, working memory refers to holding facts in mind while manipulating information, and set-shifting refers to the ability to display flexibility when there are changing rules/schedules of reinforcement in the environment (Miyake et al., 2000). The findings on the association between temporal discounting and executive functions have been somewhat mixed. Shamosh et al. (2008) reported a significant relationship between temporal discounting and working memory in a sample of university students. However, Steinberg et al. (2009) did not find a significant association between temporal discounting and executive functions in their large sample spanning 10–30 year-olds. In another study using a developmental sample, inhibition was also not found to be associated with delay discounting (Lamm et al., 2006).

Thinking dispositions are another separable domain from intelligence and executive functions that have been associated with rational thinking and decision-making (Stanovich, 2011). Thinking dispositions assess people’s propensities or tendencies that can facilitate reflective judgments. Individual differences in certain types of thinking dispositions have been shown to predict performance on decision-making tasks independently of cognitive abilities (Stanovich, 2009, 2011). Steinberg et al. (2009) examined age differences in the self-reported tendency toward future orientation in a sample of 10 to 30-year-olds. Individuals younger than age 16 had a lower orientation to the future and also accepted smaller, more immediate rewards, as opposed to larger delayed ones. We examined two thinking dispositions that we expected would be associated with temporal discounting: persistence in thinking (the Need for Cognition Scale: Cacioppo et al., 1996) and the Consideration of Future Consequences (CFCs) Scale (Strathman et al., 1994). These dispositions have been associated with better rational thinking performance in other studies (Toplak et al., 2007; West et al., 2008), and thus we expected that endorsement of persistence in thinking and higher CFCs would be associated with more delayed choices on temporal discounting.

### Associations between Behavioral Outcomes and Temporal Discounting

There is a sizable literature to suggest that temporal discounting is associated with more risky behavior. Temporal discounting studies have shown that cigarette smokers (Bickel et al., 1999), substance abusers with gambling problems (Petry and Casarella, 1999) and heroin addicts (Kirby et al., 1999) discount the value of delayed rewards at a higher rate than do control groups. Moreover, fiscal responsibility and credit card debt have been shown to be associated with low levels of CFCs and high levels of temporal discounting (Joireman et al., 2005). We examined substance use and gambling behavior in association with temporal discounting in the current study.

### Hypotheses

In this study, we examined four different dependent measures to assess temporal discounting choices: the indifference point, *k*-values, area under the curve, and an additional variable termed the interest rate total score. We expected that temporal discounting choices would be better represented by a hyperbolic than exponential curve (Rachlin et al., 1991; Green et al., 1997; Johnson and Bickel, 2002; Myerson et al., 2003; Robles and Vargas, 2007; Steinberg et al., 2009). We expected significant associations between the four indicators of temporal discounting and that reaction times would be higher at the indifference point selection than at the other choices. The preference for a larger delayed reward was also expected to be significantly associated with higher cognitive abilities, dispositional tendencies toward more persistence in thinking and CFCs, and less substance use and gambling behavior.

## Materials and Methods

### Participants

The final sample consisted of 99 participants (37 males and 62 females) from an undergraduate university sample. The mean age of the sample was 20.72 years (SD = 2.36, range = 18–30 years of age). Participants were recruited on a university campus, and each volunteer received $15 for 1 h of participation. As inclusion criteria, participants were required to have English as a first language or have been schooled in English and must have spoken English for at least 10 years. First year undergraduate students comprised 29.3% of the sample, 32.3% were second year undergraduates, 12.1% were third year undergraduates, 18.2% were fourth year undergraduates, and 8% had graduated or were post-undergraduate continuing education students.

### Measures

#### Temporal Discounting Task

A staircase version of a temporal discounting choice task adapted from Rachlin et al. (1991) was used in this study. This task was administered on a computer using the program Media Lab. This task involved making several hypothetical choices between an immediate reward and a delayed fixed reward. There were five delay periods (1 month, 1, 5, 10, and 25 years) crossed with two reward magnitudes ($100 and $10,000), both within-subject factors. The immediate variable reward changed in a sequential staircase manner by factors of 10. For example, in the $100 reward magnitude block, the immediate reward changed by $10 increments ($10, $20, $30, $40, $50, $60, $70, $80, $90, and $100). Each participant made a total of 100 choices (2 reward magnitudes × 5 delay periods × 10 trials at each reward magnitude and delay period)^1^. For the temporal discounting task, our criteria to determine the indifference point was a switch followed by two consistent choices after the switch (based on Hurst et al., 2010). We did not identify any non-systematic responders on the temporal discounting task. It should also be noted that the participants were individually tested with an experimenter, and the experimenter would have clarified any unusual responding during the testing session. The reaction time for each choice in the temporal discounting task was also collected.

Four sets of dependent variables were derived from this task. First, the temporal discounting task had a *k*-value, an individual fitted parameter that can be thought of as sensitivity to delay. When the value of *k* is small, the individual is less sensitive to delay, and shows less discounting in response to delay. When the value of *k* is large, it means that the individual is very sensitive to the delay period, and this translates to higher rates of discounting in response to delay. Since *k*-values represent a rate of discounting over time, they can only be calculated for each reward magnitude and not individual delay periods. There were three *k*-value dependent measures on this task: the mean *k*-value at the $100 reward magnitude, the mean *k*-value at the $10,000 reward magnitude, and the mean *k*-value across reward magnitudes (correlation across two magnitudes, *r* = 0.46, *p* < 0.0001). The *k*-values were skewed and not normal and could not be statistically transformed. A smaller *k*-value was associated with less discounting and a preference to wait for larger delayed reward.

The area under the curve was calculated by normalizing each delay and subjective value for each data point (Myerson et al., 2001). This was accomplished by making the delay and subjective values proportions of the maximum delay and maximum subjective values. The normalized values were then used as *x* (period of delay) and *y* (mean indifference point) coordinates to construct a graph of the discounting data. Vertical lines were drawn under each of the data points to the x-axis creating a series of trapezoids. The area of each of the trapezoids was equal to (x2 – x1)[(y1 + y2)/2]. The values of *x* were the successive delays and the *y*-values were the subjective values associated with each delay (note the first trapezoid had an x1 and y1 defined by 0.0 and 1.0). The area under the discounting function was equal to the sum of the areas of these trapezoids. Since all the values are normalized, the area under the curve can vary between 0 (steepest discounting) and 1 (no discounting). There were 13 dependent measures available for the area of the curve dependent measure: an estimate at each delay period by reward magnitude (10 values), an estimate at each reward magnitude (two values; correlation across these two reward magnitudes was *r* = 0.62, *p* < 0.0001), and an overall mean estimate. The steeper the function, the less area that is under the curve. Therefore, smaller areas under the curve represented more discounting and a preference for small immediate rewards over larger delayed rewards. Larger areas under the curve represented less discounting and a preference to wait for large delayed rewards over small immediate ones. The area under the curve measures were highly skewed and could not be normalized.

The third set of dependent measures from this task was the mean indifference point at each of the five delay periods within each of the two reward magnitudes. An overall mean indifference point was also derived for each of the two reward magnitudes, and an average indifference point was derived based on summing standardized *z*-scores of the mean indifference points at each reward magnitude (correlation across two reward magnitudes, *r* = 0.68, *p* < 0.0001), resulting in 13 indifference point scores. The overall mean score was normally distributed. The indifference points were also used for the curve fitting analyses. A larger overall indifference point represents a preference to wait for a larger delayed reward over a smaller immediate reward.

The fourth measure, the interest rate total score, was based on selecting items that met a cut-off in terms of simple rate of return on an annual basis. The simple rate of return was calculated for each of the 100 trials, and those items that were at 40% or higher were selected to derive these scores. Selecting a cut-off this high also makes salient the poor judgment to decline this return^2^. We examined the data to determine the frequencies of now versus later choices, and we decided based on a yearly interest rate that would denote variability between responders for each choice across each delay period and within each amount of delay. Specifically, at the $100 amount across the different delays, 31–97% of responders chose to wait, and at the $10000 amount across the different delays, 51–97% of responders chose to wait. We determined that a 40% cut-off would permit inclusion of at least one item from each set of choices with some variability in the proportion of now versus later choices, with the exception of the 25 years delay period. On these items, participants were credited with one point for choosing the delayed option. Participants received a score of 0 if they chose the now option on such an item. For the 1 month choices, nine out of 10 items met this cut-off. For the 1 year choices, seven out of 10 items met this cut-off; for the 5 years choices, three out of 10 items met this cut-off; for the 10 years choices, two out of 10 items met this cut-off; and no items met this cut-off for the 25 years period of delay. This yielded a total of 11 interest rate scores: eight scores for each delay period at each reward magnitude, two overall total scores for each reward magnitude (*r* = 0.72, *p* < 0.0001) and an overall total based on all 42 items. The interest rate score was skewed and could not be statistically transformed. A higher score indicated better choices on this index.

For the reaction time data, an outlier analysis was conducted with the reaction time scores on the temporal discounting task; no outliers were identified. Mean reaction time on the switch point trial was compared with the mean of the remaining trials in each block. Reaction time comparisons were available for each delay period at each amount of reward level.

### Intellectual Ability and Executive Function Measures

#### Wechsler Abbreviated Scales of Intelligence (WASI; Wechsler, 1999)

The Vocabulary and Matrix Reasoning subtests from the Wechsler Abbreviated Scales of Intelligence (WASI) were used. The Vocabulary subtest was used as an estimate of verbal ability. The Matrix Reasoning was used as an estimate of non-verbal ability. The estimated full-scale mean score based on age norms for the sample was 106.84 (SD = 10.81, range: 82–130). The mean raw score on the Vocabulary subtest was 58.75 (SD = 7.3, range = 32–73), and the mean raw score of the Matrix Reasoning subtest was 27.24 (SD = 3.89, range = 17–33). The raw scores for the Vocabulary and Matrix Reasoning subtests were converted into *z*-scores and summed to create a composite measure of intellectual ability that was not relativized to age; this score was used in the analyses. A higher score indicated higher intellectual ability.

#### Executive Function Composite

Three measures were used to index executive functions: the Stroop Test (Stroop, 1935), the Trail Making Test (Reitan, 1958), and the Paced Serial Addition Task (PASAT; Gronwall, 1977).

The Stroop Test was used to assess interference control, a type of inhibition (Friedman and Miyake, 2004). Interference control refers to the ability to filter out irrelevant information and to select relevant information. There were three different conditions in the Stroop Test: a word reading condition, a color naming condition, and an interference condition. For each condition, participants had a practice trial prior to the actual trial. In the word reading condition, participants were presented with a chart of 48 words that named four colors (red, green, blue, yellow) presented in a matrix of six columns and eight rows. Participants were asked to read the words as quickly as possible without making any errors. In the color naming condition, participants were presented with a chart of 48 patches of color (red, green, blue, yellow) presented in a matrix of six colors and eight rows. Participants were asked to name the colors as quickly as possible without making any errors. In the interference condition, participants were presented with a chart of 48 words presented in a matrix of six columns and eight rows. In this condition, the color naming words (red, green, blue, yellow) appeared in a different color (red, green, blue, yellow) than the color the word named. For example, the word “red” appeared in the color yellow. Participants were asked to name the color as quickly as possible without making any errors. The interference condition was the most difficult of the three conditions, as participants needed to inhibit the competing modality, that is, naming the words. The dependent measure was the total naming time for the interference condition minus the total naming time for the color naming condition (Strauss et al., 2006). The mean interference score was 19.13 (SD = 7.09, range = 1.98–40.01). The scores were transformed to *z*-scores. The *z*-scores were reflected so that higher scores indicated better ability to inhibit (less interference).

The Trail Making Test (Reitan, 1958) consisted of two parts, Part A and Part B. Participants completed practice items for both Part A and Part B. Part A required participants to connect with a pencil line 25 numbered circles in numeric order. Part B consisted of 13 numbered and 12 lettered circles, and the participant was instructed to alternate between letters (i.e., 1 to A, A to 2, 2 to B) until all of the circled numbers and letters were exhausted. The dependent measure was the total completion time on Part B, as this part of the task required participants to “shift set” between numbers and letters. Mean completion time was 57.28 s (SD = 18.61 s, range = 22.78–115.75). Reaction time was significantly skewed and a square root transformation was used. The scores were transformed to *z*-scores, and these scores were reflected so that higher scores indicated better set-shifting ability.

The PASAT (Gronwall, 1977) was used as a measure of working memory. It is a serial-addition task used to assess capacity and rate of information processing, and sustained and divided attention (Spreen and Strauss, 1998). It has been conceptualized as a measure of working memory that relies on attentional capacity and processing speed (Gonzalez et al., 2006). In this task, a computer was used to serially present single digits at a rate of every 3 s (Trial 1) and every 2 s (Trial 2). A practice trial preceded each of the actual trials. In each trial, the participant added each new digit to the one immediately prior to it. The percent correct from each trial was averaged across both trials. The mean overall percent correct was 68.26% (SD = 0.17, range = 0.33 to 0.97). The overall percent correct score was transformed into a *z*-score. A higher score indicated better performance.

An executive function composite was calculated by summing the *z*-scores of the three tasks. All of these measures were significantly correlated with each other (*r* = 0.31, *p* < 0.01 to *r* = 0.42, *p* < 0.0001). A higher score indicated better executive function performance.

### Thinking Dispositions

Dispositions were measured using two different scales, intermixed in a single questionnaire: the Need for Cognition Scale and the CFCs Scale. The response format for each item in the questionnaire was: Strongly Agree (6), Moderately Agree (5), Slightly Agree (4), Slightly Disagree (3), Moderately Disagree (2), and Strongly Disagree (1).

#### Need for Cognition Scale (Cacioppo et al., 1996)

The 18-item Need for Cognition Scale was used in this study. Sample items include: “The notion of thinking abstractly is appealing to me,” and “I would prefer a task that is intellectual, difficult, and important to one that is somewhat important but does not require much thought.” The mean score on this scale was 70.73 (SD = 12.87, range = 40–90). The Cronbach’s alpha on this scale was 0.86. Higher scores on the scale indicate more persistence and engagement in thinking.

#### Consideration of Future Consequences Scale (CFC; Strathman et al., 1994)

The CFC is a 12-item scale that measures the extent to which individuals consider distant outcomes when choosing their present behavior. A sample item from the scale is: “I only act to satisfy immediate concerns, figuring the future will take care of itself” (reverse scored). The mean score on this scale was 50.51 (SD = 8.29, range = 30–71). The Cronbach’s alpha on this scale was 0.79. Higher scores indicate more CFCs of behaviors.

### Behavioral Correlates Related to Negative Outcomes

#### Problem Gambling Severity Index (PGSI; Ferris and Wynne, 2001)

The PGSI consists of nine items that asked participants to rate the frequency of gambling related behavior in the past 12 months. Items consisted of questions such as: “Have you bet more than you could really afford to lose?” and “Has your gambling caused any financial problems for you or your household?” Participants were asked to rate the frequency of such items on a scale of Never (1), Sometimes (2), Most of the time (3), and Almost Always (4). There were two outliers in the data that were winsorized. Higher scores on the PGSI indicated a greater risk of gambling behavior.

#### Drug and Alcohol Problem Reporting

Each participant made ratings on a series of four questions to determine if drinking alcohol and using drugs creates problems for the individual. The items were: (1) Drinking alcohol is a problem for me, (2) Drinking alcohol has created problems between me and my partners and friends, (3) Doing illegal drugs is a problem for me, and (4) Doing illegal drugs has created problems between me and my partners and friends. Each of these statements was rated on a scale of: Strongly Disagree (1), Moderately Disagree (2), Slightly Disagree (3), Slightly Agree (4), Moderately Agree (5), and Strongly Agree (6). Higher scores indicated more problems associated with drug and alcohol use.

Our sample was relatively low risk, endorsing little gambling (*M* = 9.68, SD = 1.88, with a potential range of scores between 4 and 36) and drug and alcohol behaviors (*M* = 6.28, SD = 3.83, with a potential range of 4–24). These two measures were significantly correlated, *r* = 0.49, *p* < 0.0001, and thus a composite score was derived. We called this measure the drug and gambling composite, and a higher score indicated more drug and gambling problem behaviors.

### Statistical Analyses

We used a repeated measures design to assess the impact of length of delay (1 month, 1, 5, 10, and 25 years) and amount of reward ($100 versus $10000) on temporal discounting choices. A non-linear regression was conducted to see whether a hyperbolic function described the data better than an exponential curve by examining the proportion of variance explained by each. The non-linear regressions and curve fitting were conducted in the program GraphPad Prism. Reaction times at indifference point and non-indifference point choices were compared using non-parametric tests. Correlation analyses were conducted to determine the associations between temporal discounting, intelligence, executive functions, thinking dispositions measures, and our outcome measure. A regression analysis was conducted to determine whether intelligence, executive function, and thinking dispositions measures significantly predicted temporal discounting. The indifference point was used for the repeated measures and multiple regression analyses since it was the only normally distributed temporal discounting measure.

## Results

### Temporal Discounting: Length of Delay and Amount of Reward

The dependent measures derived from the temporal discounting task are displayed in **Table 1**. All of the temporal discounting measures have values at different delay periods and at reward magnitudes, except for the *k*-value which is derived based on compiling choices from the five different delay periods, thus resulting in two dependent measures for the *k*-values. A multivariate repeated measures analysis was conducted with the indifference point scores, with five levels of length of delay and two levels of reward magnitude. The analysis indicated a significant main effect of length of delay, *F*(4,95) = 75.66, *p* < 0.0001; η^2^ = 0.76, a significant main effect of reward magnitude, *F*(1,98) = 614.80, *p* < 0.0001; η^2^ = 0.86, and a significant interaction between length of delay and reward magnitude, *F*(4,95) = 73.63, *p* < 0.0001; η^2^ = 0.76. This interaction indicates that temporal discounting decreases at an accelerated rate in the $100 reward magnitude relative to the $10000 reward magnitude. The same pattern was apparent with the area under the curve and interest rate scores. A non-parametric analysis with the Wilcoxon Signed-Rank test indicated that the *k*-value for the reward magnitude of $100 was significantly higher than the reward magnitude for $1000, *z* = -2.73, *p* = 0.006. This analysis suggests that participants were more sensitive to the delay periods at the $100 reward magnitude compared to the $1000 reward magnitude.

**Table 1:** Means and standard deviations for temporal discounting dependent measures.

	Area under the curve		*k*-value		Indifference point		Interest rate score^2^	
Delay	*M*	SD	*M*	SD	*M*	SD	*M*	SD
**$100**								
1 month	<0.01	<0.01	–	–	83.23	25.31	0.81	0.28
1 year	0.03	0.01	–	–	52.53	32.52	0.57	0.39
5 years	0.07	0.05	–	–	33.84	30.40	0.45	0.46
10 years	0.06	0.06	–	–	27.17	27.11	0.38	0.47
25 years	0.15	0.15	–	–	22.53	24.92	-^3^	-^3^
Overall	0.31	0.24	0.26	0.66	43.86	22.23	0.64	0.29
**$10,000**								
1 month	<0.01	<0.01	–	–	9000.00	1916.63	0.88	0.22
1 year	0.03	0.01	–	–	7343.43	2868.41	0.78	0.30
5 years	0.09	0.04	–	–	3444.44	3058.02	0.73	0.43
10 years	0.08	0.06	–	–	4606.06	3349.74	0.67	0.46
25 years	0.22	0.18	–	–	3444.44	3058.02	-^3^	-^3^
Overall	0.44	0.27	0.13	0.51	5567.68	2231.63	0.81	0.24
**Average**	0.37	0.23	0.19	0.50	-^1^	-^1^	0.72	0.25

*(1) Since these scores would be composed of averaging across different reward magnitudes, they were omitted here. Elsewhere, average indifference points are computed based on standardized z-scores*.

*(2) The Interest Rate Score is presented as a percent of points possible*.

*(3) The Interest Rate Score is based on interest rate returns above 40% annually, at 25 years there was no interest rate that was at this level therefore there is no data reported*.

### Temporal Discounting: Hyperbolic versus Exponential Functions

Non-linear regressions were conducted to determine whether a hyperbolic equation or an exponential equation best described the indifference points plotted against the delay period. Four non-linear regressions for each participant were completed in total. There were two reward magnitudes, $100 and $10,000, and two non-linear regressions, hyperbolic and exponential. In the $100 reward magnitude, the mean percent of variance accounted for by the hyperbolic equation was 79.50%, compared to the mean percent of variance accounted for by the exponential equation was 73.48%. In the $10,000 reward magnitude, the mean percent of variance accounted for by the hyperbolic equation was 69.90%, compared to the mean percent of variance accounted for by the exponential equation was 65.61%. Therefore, although both equations accounted for most of the data, the hyperbolic function accounted for more of the variance than the exponential function.

### Temporal Discounting Choices Reaction Times

The reaction times for each choice on the temporal discounting task were divided into reaction times at the indifference point and the mean of the reaction times on the choices on the remaining trials. These reaction times in milliseconds are shown in **Table 2**. The reaction times are displayed for each delay period across each reward magnitude. All of the reaction times were significantly positively skewed, and transformations did not eliminate the extreme skewness of these variables. Our key comparisons were the reaction time at the indifference point choice compared with the other choices at each reward level and delay period. Thus, we conducted non-parametric analyses that are not impacted by non-normal or skewed distributions.

**Table 2:** Mean reaction time (milliseconds) at the indifference point and mean reaction time on non-indifference point temporal discounting choices.

Delay	Indifference point reaction time (ms)		Mean reaction time without indifference point (ms)
	*M*	SD	*M*	SD
**$100**
1 month	5920.16	5071.23	2775.08	1596.05
1 year	3879.94	5320.22	1776.55	1300.34
5 years	2617.29	2724.40	1249.51	759.14
10 years	2826.95	2914.93	982.03	625.71
25 years	2509.17	2941.91	949.45	862.87
**$10000**
1 month	3833.71	3989.50	1631.81	1124.76
1 year	3125.90	3706.64	1254.14	949.48
5 years	2400.16	3671.80	1236.76	1009.03
10 years	2740.30	3485.56	1112.30	782.21
25 years	2400.16	3671.80	1068.86	833.56
**Average**	3225.37	1900.56	1403.65	618.37

A Wilcoxon Signed-Rank test revealed statistically significant differences between the response at the indifference point and the other responses at each reward level and delay period, ranging from *z* = -3.20, *p* < 0.001 to *z* = -5.86, *p* < 0.0001. An overall mean reaction time score was derived for the indifference point choice and the non-indifference point choices across all reward levels and delay periods; this analysis also indicated a significant difference, *z* = -8.49, *p* < 0.0001. Across all of these analyses, participants displayed a longer reaction time when they made their choice on the indifference point trial than on the non-indifference point trials.

### Associations between Temporal Discounting Dependent Measures, Intelligence, Executive Functions, Dispositions, and the Drug and Gambling Composite

**Table 3** displays the correlations between the four total temporal discounting dependent measures, reaction time at the indifference point on the temporal discounting task, intelligence, executive functions, dispositions, and the drug and gambling composite. As the *k*-value, area under the curve, interest rate total score, and the drug and gambling composite were significantly skewed, non-parametric Spearman’s Rank Order correlations were used for these measures in the analyses. All of the different temporal discounting dependent measures were significantly intercorrelated, with correlations ranging from -0.75, *p* < 0.0001 to 0.96, *p* < 0.0001. The correlations with the *k*-value are negative because large *k*-values represent more discounting and a preference for immediate smaller reward, which is in the opposite direction of the other temporal discounting measures. Therefore, the more sensitivity to delay, the higher the *k*-value and the lower the area under the curve, indifference point, and interest rate score values.

**Table 3:** Correlations between temporal discounting dependent measures, cognitive abilities, dispositions, and outcome variables.

	1	2	3	4	5	6	7	8	9	10
**Temporal discounting**										
(1) Indifference point	1									
(2) Area under the curve^1^	0.96^∗∗∗^	1								
(3) *k*-value^1^	-0.77^∗∗^	-0.81^∗∗∗^	1							
(4) Interest rate percent score^1^	0.93^∗∗∗^	0.85^∗∗∗^	-0.75^∗∗^	1						
(5) Reaction time at switch point	0.09	0.15	-0.23^∗^	0.06	1					
**Cognitive ability**										
(6) Intelligence composite *z*-score	0.27^∗∗^	0.29^∗∗^	-0.30^∗∗^	0.27^∗∗^	0.24^∗^	1				
(7) Executive function composite *z*-score	0.22^∗^	0.27^∗∗^	-0.18	0.27^∗∗^	0.07	0.45^∗∗∗^	1			
**Thinking dispositions**										
(8) Consideration of future consequences total score	0.31^∗∗^	0.33^∗∗^	-0.32^∗∗^	0.33^∗∗^	0.27^∗∗^	0.42^∗∗∗^	0.13	1		
(9) Need for cognition total score	0.14	0.15	-0.24^∗^	0.19	0.25^∗^	0.43^∗∗∗^	0.23^∗^	0.51^∗∗∗^	1	
**Outcome**										
(10) Drug and gambling composite^1^	-0.21^∗^	-0.16	0.10	-0.18	-0.03	-0.11	-0.14	-0.11	-0.13	1

*^1^Spearman’s Rank Order correlations were used with the area under the curve, k-value, interest rate percent score, drug, and gambling composite measures as they were highly skewed*.

*^∗^p < 0.05; ^∗∗^p < 0.01; ^∗∗∗^p < 0.001*.

All four temporal discounting measures were consistently significantly associated with intelligence, in the expected direction (ranging from *r* = 0.27, *p* < 0.01 to *r* = -0.30, *p* < 0.001). The indifference point, area under the curve, and interest rate score were significantly associated with the executive function composite also in the expected direction, ranging from *r* = 0.22, *p* < 0.05 to *r* = 0.27, *p* < 0.01^3^. The CFC was significantly associated with all four temporal discounting measures (ranging from *r* = -0.32, *p* < 0.01 to *r* = 0.33, *p* < 0.01). Only one significant association between temporal discounting and the Need for Cognition Scale was obtained with the *k*-value. The choice to wait for a larger delayed reward was associated with higher intelligence, better executive functions, and more CFCs.

The indifference point was significantly associated with the drug and gambling composite, *r* = -0.21, *p* < 0.05. The other associations between the temporal discounting measures and the outcome variable were in the expected direction, but did not reach statistical significance. Overall, the choice to wait for the larger delayed reward was associated with less substance use and less gambling behavior.

The mean reaction time at the indifference point across all reward levels and delays was significantly associated with the *k*-value in the expected direction, but not with any of the other temporal discounting dependent measures. Then, this reaction time measure was significantly associated with some of the individual difference variables, including intelligence, CFCs, and need for cognition. Longer reaction times were associated with higher intellectual abilities, more CFCs, and higher persistence in thinking.

### Hierarchical Regression Analyses Predicting Temporal Discounting

Given the correlation analyses presented in **Table 3**, it is clear that intelligence, executive function, and CFCs all account for a significant amount of variance in the indifference point score, *r*^2^ = 0.07, 0.05, and 0.10 respectively. Since executive function, intelligence and CFCs are somewhat intercorrelated, we wanted to determine how much of the variance was accounted for in the indifference point while controlling for the other predictors. Specifically, we wanted to examine the linear combination of intelligence and executive function in predicting the indifference point, and we also wanted to examine whether the inclusion of CFCs explained a significant amount of variance over and above that accounted for by intelligence and executive functions. The results of this hierarchical regression are presented in **Table 4**. The hierarchical regression was estimated using ordinary least squares regression.

**Table 4:** Hierarchical regression analyses of the indifference point predicted by two models.

Variable	*B*	SE*(B)*	95% CI	*t(df)*	sr2
**Model 1**				
Intelligence composite *z*-score	0.10	0.05	0.00, 0.20	1.98 (96)^∗^	0.04
Executive functions composite *z*-score	0.04	0.03	-0.03, 0.11	1.72 (96)	0.01
**Model 2**				
Executive functions composite *z*-score	0.05	0.06	-0.06, 0.16	0.88 (95)	0.01
Intelligence composite *z*-score	0.05	0.03	-0.02, 0.11	1.37 (95)	0.02
Consideration of future consequences total score	0.02	0.01	0.00, 0.04	2.36 (95)^∗^	0.05

*^∗^p < 0.05*.

*R^2^ for Model 1 = 0.09, F (2,96) = 4.6, p = 0.01, ΔR^2^ for Model 2 = 0.05, ΔF (1,95) = 5.58, p = 0.02*.

The linear combination of intelligence and executive function contained in Model 1 accounted for a significant amount of variance in the indifference point. Controlling for executive function, intelligence was a significant predictor of the indifference point, however, controlling for intelligence, executive function was no longer a significant predictor in the model. This finding tells us that intelligence and executive function share a large amount of variance in the predictor variable and therefore when both variables are added to the regression model, only intelligence explains a significant amount of variance over and above that shared with executive function.

Next, the CFCs scale was included in Model 2. Indeed, the linear combination of variables in Model 2 accounted for a significant amount of variance in the indifference point. Controlling for the other variables, the CFCs scale was significantly related to temporal discounting, such that participants with more CFCs were more willing to wait for delayed reward. Controlling for executive function and the CFCs scale rendered intelligence a non-significant predictor in this model. Controlling for intelligence and the CFCs scale left executive functions non-significant in this model. Regression Model 2 also predicted a significant amount of variance in the indifference point over and above the variance accounted for by Model 1 and thus the inclusion of the CFCs provided a better model.

## Discussion

All of the temporal discounting measures (area under the curve, *k*-value, indifference point, and interest rate total score) were significantly correlated with one another and the preference to wait for a larger later reward was consistently associated with higher intelligence, executive functions, and more CFCs. We also found a magnitude effect, such that higher reward magnitudes were associated with less temporal discounting. There was also an interaction between delay period and amount of reward, suggesting accelerated temporal discounting for lower amounts. We found these magnitude and delay effects for the indifference point, and similar patterns were apparent for the area under the curve and the interest rate total score measures. When we examined the curve of the function created by the temporal discounting data, we found that a hyperbolic equation described the data better than an exponential equation. Moreover, when making the temporal discounting choices, participants had significantly longer reaction times on the indifference point choice than on all of the other choices. The indifference point, in particular, was significantly associated with alcohol, drug, and gambling problems. Finally, hierarchical regression of intelligence, executive functions, and CFCs significantly predicted temporal discounting choices on the indifference point dependent measure.

### Assessing Temporal Discounting using a Single Indicator

The changes in temporal discounting that occur over time and across reward magnitudes were replicated in the present research findings. As delay increased, people preferred the more immediate reward as opposed to the larger delayed reward. These findings are consistent with the finding that the amount of the immediate reward that is subjectively equivalent to the delayed reward, decreased with delay (Green et al., 1997; Steinberg et al., 2009). There were also significant differences between each of the $100 and $10,000 magnitude reward blocks. There was less discounting of the delayed reward in the $10,000 block even though the amount of the immediate and delayed reward was proportional to that in the $100 block. Green et al. (1997) found that the rate of discounting of the delayed reward decreased in a negatively accelerating fashion from $100 to $25,000 reward magnitude blocks. Thus, as the reward magnitude increased, people were more likely to wait for delayed reward.

The indifference points were fit with non-linear regressions to both hyperbolic and exponential discounting functions. The hyperbolic discounting function accounted for more variance in the indifference point or subjective value as predicted from delay than did the exponential function. This is consistent with a body of research that has compared hyperbolic discounting functions to exponential ones, and has found that even though both models predict a large amount of variance in the indifference point or subjective value of the delayed reward, a hyperbolic function accounts for slightly more variance and is therefore a better model (Rachlin et al., 1991; Green et al., 1997; Johnson and Bickel, 2002; Myerson et al., 2003; Robles and Vargas, 2007; Steinberg et al., 2009).

We examined a further index to assess temporal discounting choices, called the interest rate total score. This measure demonstrated the same convergent patterns as the *k*-value, area under the curve, and indifference points. The purpose of this measure was to select a subset of items where participants should prefer the delayed option, where the expected utility is arguably better if one waits, making this measure a better indicator of decision-making performance. However, this measure was also skewed, similar to the *k*-value and area under the curve. The skewed distribution of the interest rate score was, however, different from the skewed distribution of the *k*-value and area under the curve measures. The *k*-value and area under the curve measures were positively skewed, reflecting a sudden hyperbolic decrease in discounting over delay period. Alternatively, the interest rate total score had a negative skew, due to the fact that there was very little variability in some of the temporal discounting choices on the other side of the distribution. For example, for the “$10 now” versus “$100 in a month” choice, three participants chose now and 96 participants chose later demonstrating that virtually all of the participants preferred to wait for the large amount. This item was a poor discriminator in our sample, and contributed to the negative skew in the distribution of the total interest rate score. However, one could design temporal discounting items with a more narrow range of discriminating items to further differentiate better judgments and choices and to derive a normally distributed measure to use for further assessments with individual difference measures. Overall, our interest rate total score measure performed similarly to the other temporal discounting indicators, including a similar pattern of associations with our individual difference measures. The basis of this measure is to differentiate poor from good choices based on an interest rate cut-off score. Thus, the interest rate total score will be a useful indicator to assess temporal discounting as a measure of rational thinking and decision-making competence (Toplak et al., 2014).

The temporal discounting task was assessed with four different dependent measures; indifference point, area under the curve, *k*-values, and an interest rate total score. The correlations ranged from 0.75, *p* < 0.0001 to 0.96, *p* < 0.0001 suggesting that these variables are largely redundant and may serve as proxies for one another. All of these dependent measures were significantly and strongly associated with each other. Since these measures are highly associated, it is acceptable to interpret results from the measures in similar ways. Myerson et al. (2001) suggested that the area under the curve be used instead of the *k*-value as an index of discounting since the *k*-value has theoretical assumptions based on the line of best fit from the non-linear regression using the hyperbolic function. Another major difference between these measures is that only the indifference point measure was normally distributed and not skewed, making it a good candidate for studies examining associations with other individual difference measures using parametric models.

### The Selection of the Larger Delayed Reward is the Considered Choice

In addition to the pattern of findings obtained with respect to delay period and reward magnitude, our analyses examining reaction time differences, associations with cognitive abilities and executive functions, and associations with thinking dispositions provides further insights into the correlates of temporal discounting choices.

Our results indicated that the reaction time during the trial in which the participant shifted from choosing the “amount now” to choosing the “amount later,” the indifference point, was significantly higher than the reaction times for the other trials. These results indicate that participants spent longer on the indifference point trial than on all of the other trials. This is consistent with research that has found that reaction times seem to parallel the effort involved in making temporal discounting choices (Robles and Vargas, 2007). This research has found a bimodal distribution in reaction times with people taking longer on the first choice and at the indifference point. The results in the current study confirm that people do take significantly longer at the indifference point which may be due to the effort involved in overriding the default choice from the previous responses, when participants are deciding when the delayed reward is subjectively equivalent to the immediate reward. The reaction time at the indifference point was also significantly correlated with some of the individual difference variables, including intellectual abilities, CFCs, and persistence in thinking, reinforcing that the choice at the indifference point was more cognitively effortful than the other choices in the task.

The association between temporal discounting and cognitive ability measures are consistent with the finding that people with higher intelligence scores have lower rates of temporal discounting or show less discounting of future reward due to time (Shamosh and Gray, 2008; Shamosh et al., 2008). For people with higher intelligence scores, the amount of a delayed reward maintains its value, and these people are willing to wait for the delayed reward as opposed to preferring the immediate smaller reward. The relationship between the executive function composite *z*-score and the temporal discounting dependent measures was very similar, and consistent with the literature that has demonstrated a relationship with cognitive abilities (Thorell, 2007; Shamosh et al., 2008; Sjöwall et al., 2013). As the overall temporal discounting indifference point increased, CFCs increased, but no relationship was obtained with the Need for Cognition Scale. This reflects the finding that the more individuals consider distant outcomes when choosing their present behavior (Strathman et al., 1994) the more likely they are to wait for delayed reward. This parallels research that has found the same relationship between future orientation and delay discounting (Steinberg et al., 2009).

We expected that those who endorse more persistence in abstract thinking would also be more likely to wait for a larger delayed reward, as both involve engagement in analytic thought and consideration, but this was not the case on the temporal discounting dependent measures. The one exception was that reaction time at the indifference point was significantly correlated with the Need for Cognition Scale, suggesting that actual time spent on the trial requiring the most consideration was perhaps the critical variable to explain this association. Prudently discounting the future and persistence in thinking may not necessarily cohere together. To our knowledge, the need for cognition scale had not been examined with respect to temporal discounting in previous literature, thus we were less surprised that this association was not significant.

Our regression analyses indicated that a model including intelligence, executive functions, and CFCs was a significant predictor of the indifference point dependent measure. While executive functions, intelligence, and CFCs were significantly associated with temporal discounting independently, when entered into the hierarchical regression, a model including CFCs explained the most amount of variance in the indifference point score. These findings are consistent with Shamosh et al. (2008) who also found that some executive functions, such as working memory, did not explain further variance than was explained by intelligence. This suggests that the predictive power attributed to intelligence and executive functions, both cognitive abilities, may be assessing processing capacities and processing efficiency (Stanovich, 2009). Since CFCs was a significant predictor in our model, this finding suggests that thinking dispositions factor strongly into temporal discounting choices, namely general preferences toward satisfying immediate needs versus regard for the future and distant outcomes. These findings reinforce that temporal discounting choices involve processing efficiencies and capacities (intelligence and executive functions) as well as dispositional tendencies that shape temporal discounting choices. Ainslie (2001) has suggested that utility theory is inadequate for explaining temporal discounting choices, as it is not just a matter of calculating the maximal reward. In fact, our regression analyses suggest that there is shared variance between cognitive abilities and CFCs that is predicting temporal discounting choices. The findings in this study demonstrate that the selection of the larger delayed reward reflects more effortful, considered processing and a resistance to miserly information processing, and thus, is a relevant measure for taxonomies of rational thinking and decision-making (Stanovich, 2009, 2011).

### Associations between Temporal Discounting and Real World Behavioral Correlates

The temporal discounting choices were only modestly correlated with the problem reporting drug, alcohol, and gambling composite measure, with only the indifference point reaching significance. This is generally in line with other studies that have shown high rates of discounting to be associated with substance use (Bickel and Marsch, 2001) and pathological gambling (Holt et al., 2003). The modesty of our associations may be partly attributable to the fact that these risky behaviors were endorsed with low frequency in the present sample of university students. Other future directions include replication of the current findings with larger sample sizes.

## Conclusion

The current study replicated findings that temporal discounting increased as delay to reward increased, and temporal discounting decreased as reward magnitude increased. We found the same pattern of effects between four indicators of temporal discounting, and consistent relationships with individual difference measures, including intelligence, executive functions, and the dispositional tendency of CFCs. The preferred choice to wait for a larger, later reward is associated with higher intellectual abilities and executive functions and the tendency to give more consideration to future consequences. The reaction time differences indicated that it took longer to make choices at the indifference point than at the other choice points. The interest rate total score measure was a converging measure of temporal discounting that may provide an additional index to assess decision-making choices in future studies. It will be fruitful to further develop such measures that separate better choices from poor choices to help identify and ameliorate failures in temporal discounting judgments that may contribute to poor outcomes.

## Conflict of Interest Statement

The authors declare that the research was conducted in the absence of any commercial or financial relationships that could be construed as a potential conflict of interest.

## Footnotes

1 The typical administration of this task involves administering each set of trials in ascending and descending order. Administering both ascending and descending order to each participant would have doubled the number of trials, thus ascending versus descending order was treated as a between-subject manipulation to limit the number of trials to 100 for each participant. Thus, half of the participants received the trials in descending order and half received the trials in ascending order. An independent-samples *t*-test was conducted to compare the indifference point for each order. Only one of the 10 analyses was significant, indicating that indifference points were not significantly different across ascending and descending versions. Thus, the choices from each order were collapsed for the analyses. Robles and Vargas (2007) also found no differences in the degree of discounting between ascending and descending procedures.

2 We realize that this cut-off was high, given that the interest rates have hovered below 5% over the last 10 years in several Western countries (USA: 2.07%; Canada: 1.75%; Britain: 1.85% based on 10-years government bonds; The Economist, 2014). However, it is important to also note the context of this task. The actual items selected for this task were based on previously published studies of items that have been used for staircase temporal discounting tasks. The interest rates of return ranged from 1 to 10800%, which is a range that people would never experience in their everyday lives. In the context of this task, participants would be contrasting choices that vary substantially in terms of the potential amount gained within this task. Likely as a result of the context of this task, there was no discrimination between people’s choices at 5%, because almost everyone chose now.

3 We compared the correlation coefficients separately for each of the executive function variables. All of the patterns were in the same direction: higher working memory, inhibition, and set-shifting were associated with higher indifference points, area under the curve, and interest rate total score, and lower *k*-values. None of the correlations differed significantly across the different executive function tasks (range of correlation for the indifference point, area under the curve and interest rate total score: working memory: *r* = 0.19, *p* < 0.10 to 0.23, *p* < 0.05; inhibition *r* = 0.12, *ns* to 0.17, *ns*; set-shifting: *r* = 0.16, *ns* to 0.20, *p* < 0.05; Correlations for *k*-value: working memory: *r* = -0.061, *ns*; inhibition *r* = -0.16, *ns*; set-shifting: *r* = -0.11, *ns*). We conducted the same set of analyses separately with verbal and nonverbal abilities, and found the same pattern (range of correlations for the indifference point, area under the curve and interest rate total score: verbal ability: *r* = 0.14, *ns* to 0.16, *ns*; nonverbal ability: *r* = 0.26, *p* < 0.01 to 0.28, *p* < 0.001; Correlations for *k*-value: verbal ability: *r* = -0.10, *ns*; nonverbal ability: *r* = -0.32, *p* < 0.001).
